# Distinct Cortical Activation in Adolescents With Social Anxiety Disorder: An fNIRS Study

**DOI:** 10.1002/brb3.71357

**Published:** 2026-03-31

**Authors:** Huishan Liu, Jinru Zhang, Ying Niu, Weihai Huang, Qiqi Li, Zhifen Liu, Gerard Leavey, Gaizhi Li

**Affiliations:** ^1^ First Clinical Medical College Shanxi Medical University Taiyuan China; ^2^ Department of Psychiatry First Hospital of Shanxi Medical University Taiyuan China; ^3^ School of Humanities and Social Science Shanxi Medical University Taiyuan China; ^4^ College of Medical Sciences Shanxi Medical University Taiyuan China; ^5^ Bamford Centre for Mental Health & Wellbeing Ulster University Belfast United Kingdom of Great Britain and Northern Ireland

**Keywords:** adolescent, BiomarkerfNIRS, social anxiety disorder

## Abstract

**Background:**

Social anxiety disorder (SAD) is prevalent among children and adolescents, manifesting as concerns and anticipatory anxiety in social settings. However, the precise mechanism remains to be elucidated.

**Aims:**

To examine the hemodynamic response patterns in individuals with SAD using functional near‐infrared spectroscopy (fNIRS), as well as the association with severity of social anxiety.

**Methods:**

Twenty‐nine adolescents diagnosed with SAD, and 36 healthy control (HC) were included in this study. FNIRS was used to access the frontal and temporal cerebral hemodynamics, expressed as Δ β values. The screen for child anxiety‐related emotional disorders (SCARED) and the social avoidance and distress scale (SAD) were used to assess severity of social anxiety.

**Results:**

Participants with SAD exhibited significantly increased Δβ values within channels CH22, CH34, and CH40 compared to the healthy control group. Conversely, diminished Δβ magnitudes were recorded in channels CH4 and CH41 among the SAD cohort relative to the HC group. The Δ β value of CH4 was negatively correlated with the social avoidance subscale of SAD, while the Δ β value of CH40 was positively correlated with the separation anxiety subscale of SCARED.

**Conclusion:**

Adolescents diagnosed with SAD display distinct hemodynamic responses in cortical regions, suggesting that such activation patterns may represent a potential biomarker for social anxiety The different regions of the pre‐frontal cortex may play distinct roles in social avoidance and separation anxiety in adolescents with SAD.

## Graphical abstract

Using functional near‐infrared spectroscopy (fNIRS) during a verbal fluency task, adolescents with social anxiety disorder (SAD) showed distinct hemodynamic responses in frontotemporal regions compared to healthy controls. The Δ β in CH4 was negatively associated with both social avoidance and social distress scores, while the Δ β in CH40 was positively associated with separation anxiety scores.

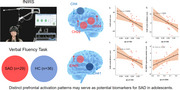

**Abbreviations**
dlPFC: dorsolateral prefrontal cortex
DMN: default mode network
DSM‐5: Diagnostic and Statistical Manual of the American Psychiatric Association (fifth edition)
FDR: False discovery rate
fMRI: functional magnetic resonance imaging
fNIRS: Functional near infrared spectroscopy
HC: healthy controls
PFC: prefrontal cortex
SAD: Social Anxiety Disorder
SCARED: Screen for child anxiety related emotional disorders
SMA: sensorimotor area
TL: temporal lobe

## 1 Introduction

Social anxiety disorder (SAD) refers to an individual's persistent worry about scrutiny or negative evaluation from others in social or performance settings, leading to avoidance of social situations or enduring intense distress or anxiety within them. It is estimated that approximately 13% of the population will meet the diagnostic criteria for SAD at some point in their lifetime, with an average estimated prevalence of 6.7‐12.1% (Fehm et al. 2005; Kessler et al. 2005) in the US sample. The prevalence of SAD among children and adolescents ranges from approximately 1% to 13% (Mohammadi et al. 2020). SAD is common during childhood and adolescence. SAD patients generally experience strong emotional fluctuations and discomfort, while also increasing the risk of school refusal, dropping out of school, poor interpersonal relationships, and susceptibility to bullying. Furthermore, it is evident that SAD frequently co‐occurs with other anxiety disorders, depression, substance abuse, and physical health disorders. Individuals diagnosed with SAD are at an elevated risk for experiencing suicidal thoughts and developing substance use disorders (Sareen et al. 2005).

Thus, establishing clear and objective diagnostic markers may help in identifying SAD patients and facilitating the development of early and precise treatment plans.

While research on SAD biomarkers has extensively covered genetic, epigenetic, endocrine, and immunological mechanisms, a growing body of neuroimaging studies has also contributed significantly to understanding its neural mechanism. Most neuroimaging studies that utilize functional magnetic resonance imaging (fMRI) have examined adult patients with SAD. Previous neuroimaging studies reported altered volumes and reactivity of the ventrolateral prefrontal cortex (vlPFC) (Auday and Pérezdgar 2019), also disrupted functional connectivity of the middle temporal gyrus (MTG) (Zhang et al. 2022), and widespread intra‐network functional network connectivity abnormalities in the default mode network (Zhang et al. 2023). Additionally, social anxiety has been associated with gray matter volume in the right middle temporal gyrus (MTG) (Wang et al. 2021), and spontaneous neural activity in the left superior frontal gyrus and right middle temporal gyrus (Ma et al. 2024). To summarize, while neuroimaging findings in adult SAD are increasingly established, studies focusing on adolescent populations remain relatively scarce, and the evidence is less consistent. Notably, recent work in general adolescent samples has linked social anxiety to structural and functional alterations in frontal‐temporal regions. For example, gray matter volume in the right MTG was associated with social anxiety, with emotional intelligence mediating this relationship (Wang et al. 2021). Separately, social intelligence was found to mediate the link between resting‐state activity in the left superior frontal gyrus and social anxiety (Ma et al. 2024). These studies highlight both the relevance of frontal‐temporal circuitry and the role of socio‐cognitive processes in adolescent social anxiety. However, there remains a gap in studies directly examining task‐evoked hemodynamic responses in clinically diagnosed adolescents with SAD compared to matched healthy controls. Given adolescents’ unique developmental needs, such as greater sensitivity to medical settings and lower discomfort tolerance, a tolerable, cost‐effective, and compliance‐friendly neuroimaging method is particularly important for identifying biomarkers in this age group.

Functional near‐infrared spectroscopy (fNIRS) is a portable, cost‐effective technique characterized by low environmental dependence, and high tolerance. The device is capable of objectively capturing the dynamic characteristics of cerebral blood flow and indirectly measuring neural activity. This is achieved by monitoring the fluctuations in the concentrations of oxygenated hemoglobin (HBO) and deoxygenated hemoglobin (HBR) in the cerebral cortex (Scholkmann et al. 2014). FNIRS is being used more in research into psychiatric disorders, such as depression and autism (Zhang et al. 2015; Scaffei et al. 2023). The Verbal Fluency Task (VFT) has been shown to engage a range of higher‐order cognitive processes, including executive control and working memory (Henry and Crawford 2004), which are essential for fluent and adaptive social communication. The VFT has been extensively validated as a robust paradigm for activating the prefrontal cortex (PFC) using fNIRS. Its simplicity and low level of discomfort also render it highly suitable for adolescent populations. The hemodynamic changes in the frontotemporal regions during VFT, frequently reported by fMRI studies, detected by fNIRS, provide an indirect reflection of brain activity status, with objective and quantitative bases for assessing the severity of clinical symptoms and cognitive function status of patients. The multi‐channel NIRS study found smaller hemodynamic changes of PFC in SAD patients compared with healthy individuals in the VFT tests (Yokoyama et al. 2015; Glassman et al. 2017). Individuals with SAD demonstrate elevated neural activity within the frontal and temporal cortical regions when exposed to social acceptance or rejection scenarios (Kir et al. 2021). Concurrently, variations in oxygenated hemoglobin levels in the left prefrontal cortex are indicative of the severity of social anxiety symptoms (Uchida and Hirao 2020).

We aimed to explore which channels show different changes in oxyhemoglobin concentration between adolescents with SAD and healthy controls, and to analyze the connection with the severity of social anxiety. We hypothesized that changes in HBO concentration in adolescents withSAD are different from those in healthy controls (HC) and can be used as potential diagnostic markers for SAD.

## 2 Methods

### 2.1 Participants

In the period between January 2024 and August 2024, a total of 29 adolescent patients diagnosed with SAD were enrolled in the study. These patients were recruited from the child and adolescent psychiatry outpatient department at the First Hospital of Shanxi Medical University. Inclusion criteria for the patients included the following:

1. Met the diagnostic criteria for SAD, without any other mental disorders diagnosis, as defined in the fifth edition of the Diagnostic and Statistical Manual of Mental Disorders (DSM‐5);
2. Aged 12–18 years old;
3. This episode marked the initial occurrence for which the individual had no prior record of receiving psychiatric interventions, including pharmacotherapy, somatic treatments, or psychological interventions;
4. Right‐handedness was demonstrated.

A total of 36 healthy volunteers were recruited from public schools in the local community through the distribution of digital flyers. Eligible participants were required to be between 12 and 18 years of age, with no limitations on gender. To screen for psychiatric conditions, all healthy controls underwent clinical evaluation using the M.I.N.I. (MINI‐International Neuropsychiatric Interview), ensuring the absence of current or historical mental health disorders including SAD.

Exclusion criteria applied to all participants, encompassing both the patient and healthy control (HC) groups, consisted of the following:

1. significant or unstable medical or neurological comorbidities;
2. a prior diagnosis of any mental disorder;
3. contraindications for fNIRS assessment, such as open cranial injuries, recent head trauma, or poorly managed seizure activity.

Furthermore, every subject underwent an independent clinical assessment conducted by two qualified child and adolescent psychiatrists.

### 2.2 Clinical Evaluation

We collected general demographic data, including gender, age and educational years. The screen for child anxiety related emotional disorders (SCARED) and social avoidance and distress scale (SAD) were used to assess the social anxiety symptoms.

The SCARED is a 41‐item instrument organized into five distinct subscales: generalized anxiety, separation anxiety, social anxiety, panic/somatic manifestations, and school avoidance. Participants were asked to report how often they experienced each symptom over the preceding three‐month period. Responses are rated on a three‐point scale consisting of “almost never” (0 points), “sometimes” (1 point), and “often” (2 points). A total score exceeding 23 points suggests the presence of clinically significant anxiety. The Chinese version of SCARED has demonstrated satisfactory test‐retest reliability and construct validity (Su et al. 2008), with Cronbach's α coefficients reported between 0.43 and 0.78 (Su et al. 2008).

The SAD consists of 28 items, of which 14 evaluate social avoidance and the other 14 measure distress in interpersonal situations. Each item is answered using a 0 to 4 point format. Total scores span from 0 to 112, where elevated scores reflect more severe levels of social avoidance and distress. The Cronbach's alpha coefficient of this scale among Chinese student populations is 0.85 (Peng et al. 2003). In this study, both scales were used in a complementary manner: the SCARED provided a broad screening of the overall anxiety profile, while the SAD scale was specifically employed to yield a detailed, disorder‐focused measurement of the two core symptom dimensions of SAD (social avoidance and social distress) for correlation with neural activity.

### 2.3 Functional Near‐Infrared Spectroscopy (fNIRS)

**FIGURE 1:**
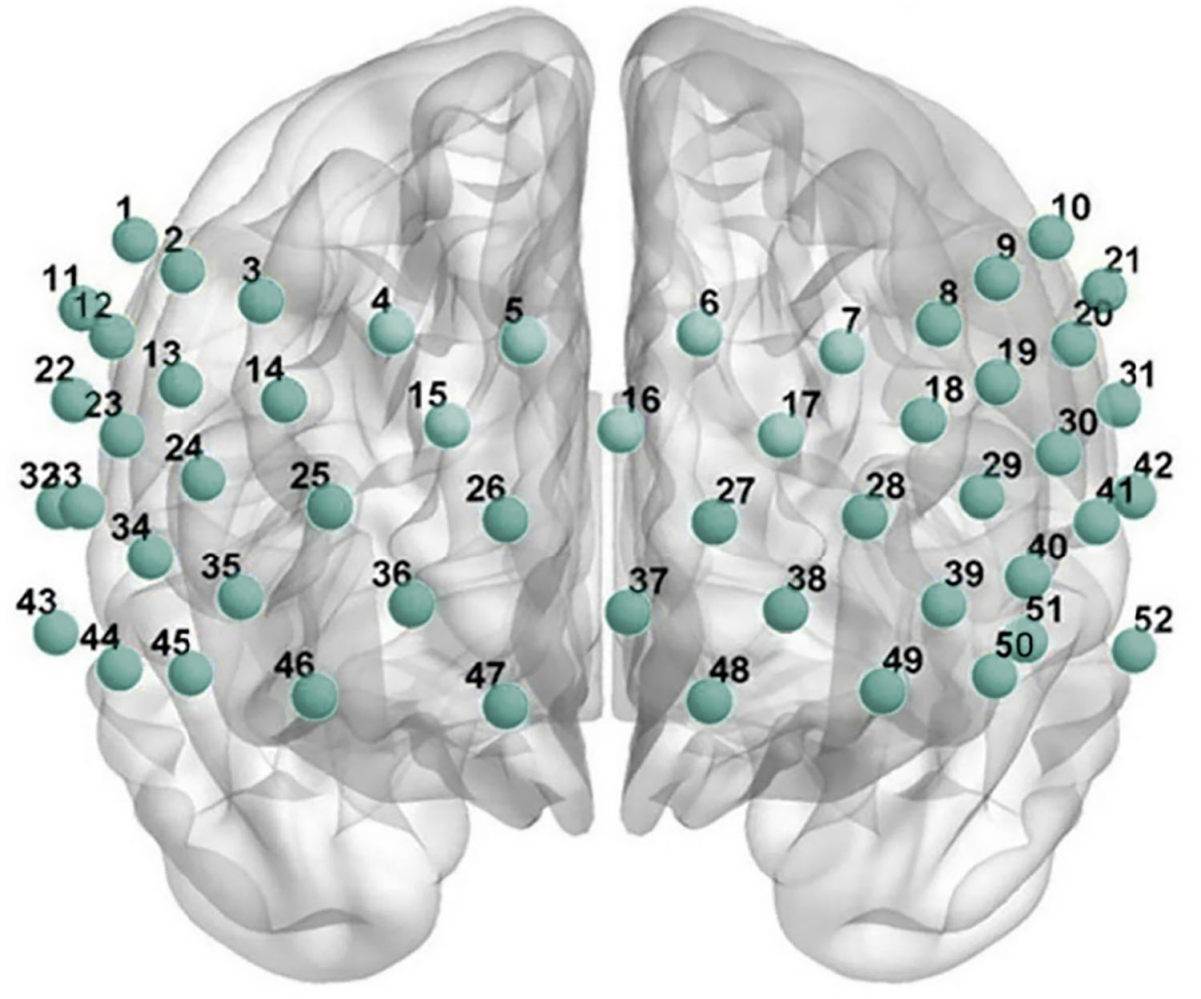
The distribution of 52 channels. The probe array was positioned according to the international 10–20 system, with the lowest row centered along the Fp1–Fp2 line. Each circle represents a measurement channel. Abbreviations: Fp, frontopolar; fNIRS, functional near‐infrared spectroscopy.

Hemodynamic responses within the prefrontal and superior temporal cortical regions were assessed using a 52‐channel functional near‐infrared spectroscopy (fNIRS) device (ETG‐4100; Hitachi Medical). The fNIRS system, with 17 transmitters and 13 receivers, provides detailed monitoring of blood flow changes in these brain regions. Relative changes in oxygenated hemoglobin (HbO) were quantified through the application of dual‐wavelength near‐infrared light (695 nm and 830 nm), utilizing the modified Beer–Lambert law for signal conversion. The probe array, composed of 17 illuminating and 16 sensing elements, was arranged in a 52‐channel layout and secured using 3 × 11 thermoplastic casings. The optodes were positioned with a fixed inter‐probe spacing of 3.0 centimeters, and hemodynamic signals were acquired at a sampling rate of 10 Hz. In accordance with the international 10–20 electroencephalography positioning standard, the probe array was aligned such that the most inferior channel was centered on the forehead along the Fp1–Fp2 axis. See Figure 1. Throughout the imaging process, participants performed the Verbal Fluency Test (VFT) to concurrently elicit cognitive activation.

### 2.4 Verbal Fluency Test (VFT)

**FIGURE 2:**
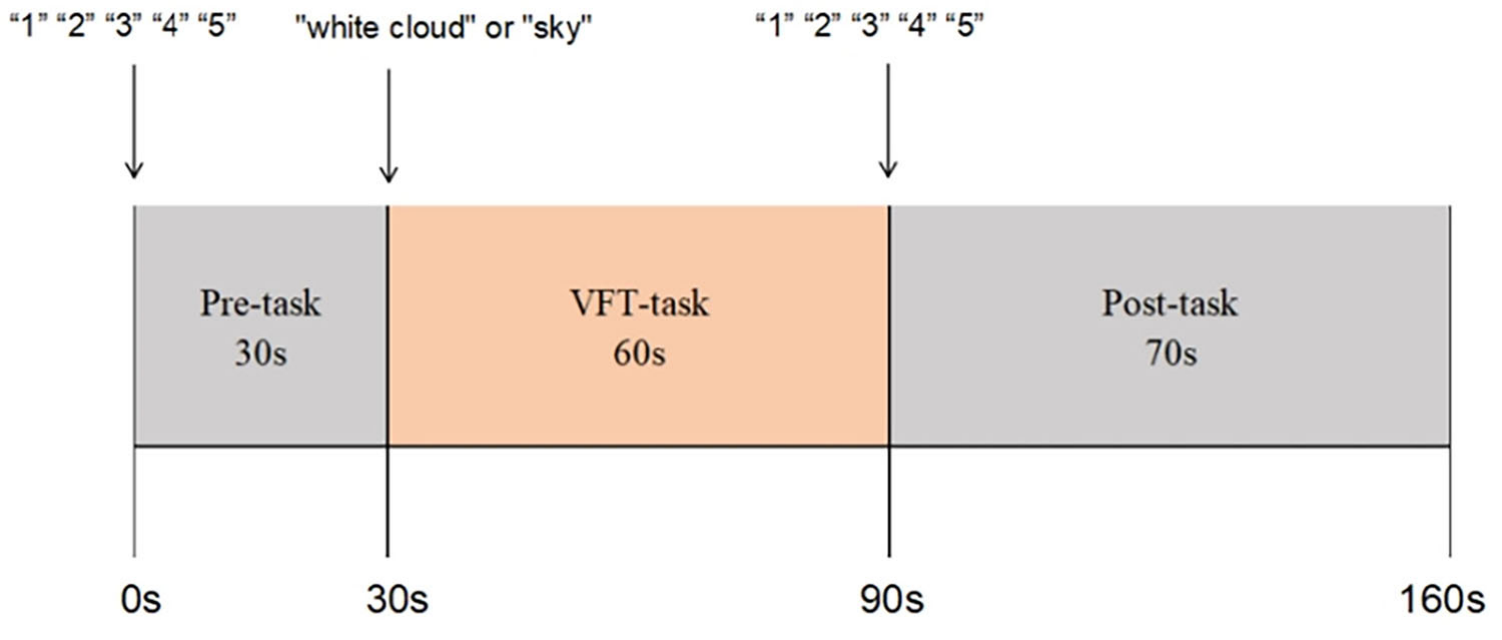
The timeline presents the stimulus during the pre‐task, task, and post‐task periods of VFT. The task consisted of a 30‐s pre‐task baseline (counting 1–5 repeatedly), a 60‐s task period (generating words for the characters “tian”, “da”, and “bai”), and a 70‐s post‐task recovery (counting 1–5). Total duration: 160 s.

The VFT is comprised of three distinct phases: the preparation phase, the task execution phase, and the recovery phase. During the preparatory phase (30 s), the participant is required to adopt a specified position, while ensuring stability and avoiding unnecessary movements. They are also instructed to focus on a cross mark on the screen, while repeatedly uttering the numbers “one, two, three, four, five” in order to establish a baseline. Subsequently, the task execution phase (60 s) is initiated, during which participants are required to verbally construct words that incorporate the specified Chinese characters “tian” (meaning sky), “da” (meaning big), and “bai” (meaning white). They received the following prompt: “Form words using ‘tian’, ‘da’, and ‘bai’. Produce at least three words; higher output is preferred.” Finally, a 70‐second recovery phase followed, in which subjects repeatedly recited the numeric sequence from one to five until the task was complete. A skilled psychiatric clinician oversees the entire 160 s process, ensuring the participant understands the task. See Figure 2.

### 2.5 fNIRS Statistical Analysis

During the VFT task, the fNIRS device collected data regarding HBO. The processing of the data was conducted utilizing MATLAB 2013b and the NIRS‐SPM toolbox.

1. Preprocessing of the acquired fNIRS signals involved convolution with a hemodynamic response function (HRF) and application of a discrete cosine transform (DCT) to attenuate low‐frequency drifts and motion‐induced artifacts.
2. A General Linear Model (GLM) was formulated using the equation Y = βX + ε, in which Y corresponds to the fNIRS‐measured oxygenated hemoglobin (HbO_2_) concentration changes, X indicates the predicted hemodynamic responses derived from the experimental paradigm, and ε signifies residual unexplained variance. The estimated coefficients β reflect the magnitude of task‐evoked cortical activation during the Verbal Fluency Test.
3. For each participant, channel‐wise β values were extracted to quantify cortical activation. The differential oxygenated hemoglobin parameter (Δβ) was computed by subtracting the baseline β value from the β value obtained during the Verbal Fluency Test (VFT) task. This derived metric served as an indicator of hemodynamic activation levels within the frontal and temporal cortical regions during task performance.

### 2.6 Data Analysis

Continuous variables were analyzed via analysis of variance (ANOVA), incorporating age, gender, and years of education as covariates to examine group differences. Categorical measures were evaluated using Chi‐square tests. To account for demographic differences, age, gender, and years of education were included as covariates in all group comparisons (ANCOVA) and correlation analyses. This statistical control aims to isolate variance specifically associated with diagnostic group while adjusting for these potential confounds. All analyses adopted a two‐tailed approach, with significance thresholds set at FDR‐corrected *p* < 0.05. For the correlation analyses between hemodynamic activity and clinical symptoms (Table 3), the FDR correction was applied across the total number of tests performed (5 channels × 9 clinical measures = 45 tests). Statistical procedures were performed using SPSS version 22.0 (SPSS Inc., Chicago, IL, USA).

## 3 Result

### 3.1 Demographic Data and Clinical Characteristics

No significant difference in gender distribution was observed between the two groups (*p* > 0.05). However, participants with SAD were significantly younger and had fewer years of education compared to the HC group (*p* < 0.001). See Table 1.

**TABLE 1:** Demographic and clinical characteristics of the participants.

	SAD (n = 29) Mean ± SD	HC (n = 36) Mean ± SD	*t/*χ^2^	*P*
Age	14.93 ± 1.69	17.08 ± 0.87	43.967	<0.001***
Gender (M:F)	7:22^a^	5:31^a^	1.121	0.290
Years of education	8.28 ± 1.69	11.08 ± 0.87	74.857	<0.001***
SCARED‐total	59.07 ± 15.16	—	—	—
SCARED‐somatization	17.34 ± 6.22	—	—	—
SCARED‐generalized anxiety	14.45 ± 3.74	—	—	—
SCARED‐separation anxiety	8.03 ± 4.07	—	—	—
SCARED‐social anxiety	12.76 ± 2.36	—	—	—
SCARED‐school avoidance	5.79 ± 1.93	—	—	—
SAD‐total	91.45 ± 12.55	—	—	—
SAD‐social avoidance	44.69 ± 6.73	—	—	—
SAD‐social distress	46.76 ± 6.51	—	—	—

a: count.

Abbreviations: HC, healthy control; SAD scale, Social Avoidance and Distress Scale. ***p < 0.001; SAD, social anxiety disorder; SCARED, Screen for Child Anxiety‐Related Emotional Disorders.

### 3.2 Δβ in HBO during the VFT

**FIGURE 3:**
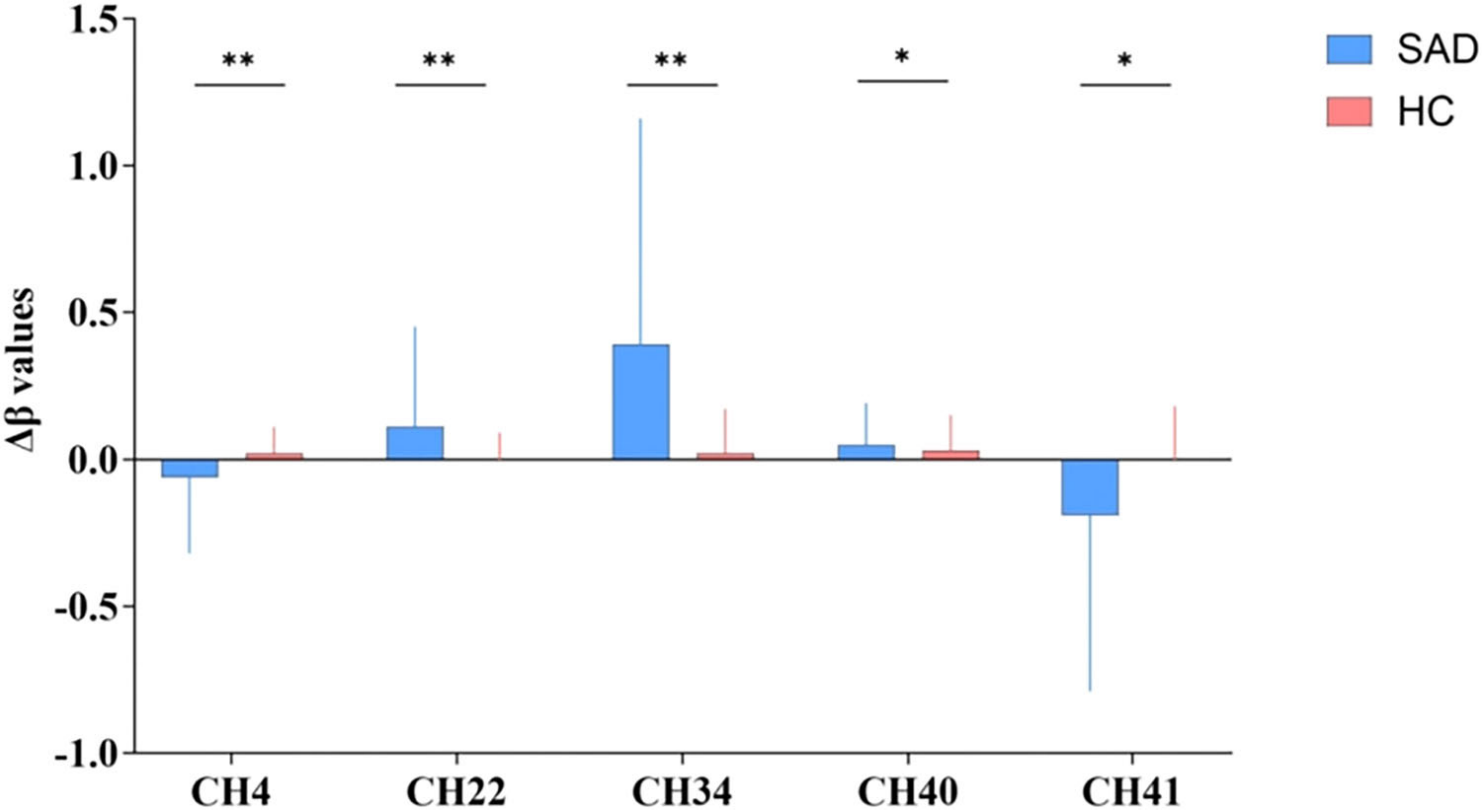
The Δβ value of channels between the two group. Bar plots show Δβ (mean ± SD) for SAD (n = 29) and HC (n = 36) groups. Δβ values reflect changes in oxygenated hemoglobin during the Verbal Fluency Task. Between‐group comparisons were performed using ANCOVA with age, sex, and education as covariates. P‐values are FDR‐corrected. *p < 0.05, **p < 0.01 (FDR‐corrected). Abbreviations: SAD, social anxiety disorder; HC, healthy control; CH, channel.

Analysis of covariance (ANCOVA), controlling for age, gender, and educational duration, demonstrated significantly decreased Δβ values in channels CH4 (*p* = 0.003) and CH41 (*p* = 0.014) among participants with SAD relative to HCs. All reported between‐group differences are thus statistically adjusted for these variables. In contrast, the SAD group exhibited increased Δβ values in channels CH22 (*p* = 0.008), CH34 (*p* = 0.001), and CH40 (*p* = 0.03) compared to the HC group. Other channels showed no statistical significance in hemodynamic activity. See Table 2; Figure 3.

**TABLE 2:** Comparison of Δβ during VFT between the two groups.

	SAD (n = 29) Mean ± SD	HC (n = 36) Mean ± SD	F	*P*
CH4	−0.06 ± 0.26	0.02 ± 0.09	9.332	0.003**
CH22	0.11 ± 0.34	0.00 ± 0.09	7.572	0.008**
CH34	0.39 ± 0.77	0.02 ± 0.15	10.692	0.001**
CH40	0.05 ± 0.14	0.03 ± 0.12	4.966	0.030*
CH41	−0.19 ± 0.60	0.0 ± 0.18	6.478	0.014*

Δβ represents task‐related change in oxygenated hemoglobin concentration. Between‐group comparisons were performed using ANCOVA with age, sex, and education as covariates. P‐values are FDR‐corrected for multiple comparisons across channels. *p < 0.05, **p < 0.01 (FDR‐corrected). Abbreviations: SAD, social anxiety disorder; HC, healthy control; CH, channel.

### 3.3 The Correlation of Clinical Symptoms and Δβ

**FIGURE 4:**
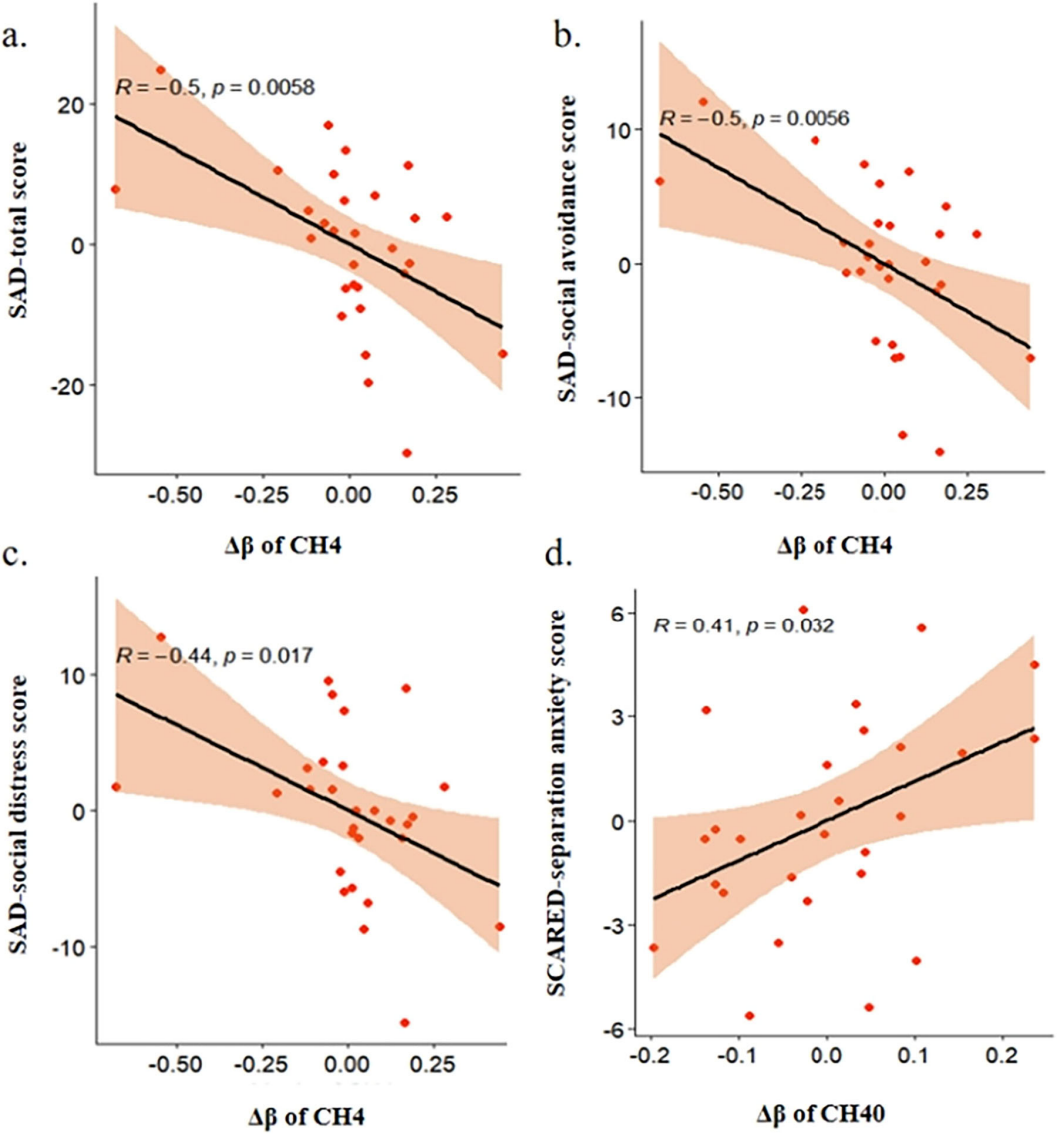
The correlation between Δ β of CH4, CH40, and clinical symptom scores. (a) Correlation between Δβ in CH4 and SAD scale total score. (b) Correlation between Δβ in CH4 and SAD social avoidance subscore. (c) Correlation between Δβ in CH4 and SAD social distress subscore. (d) Correlation between Δβ in CH40 and SCARED separation anxiety subscore. Solid lines indicate linear regression fit; shaded areas represent 95% confidence intervals. Correlation coefficients (r) and FDR‐corrected p‐values (controlling for age, sex, and education) are displayed on each plot. Abbreviations: SAD scale, Social Avoidance and Distress Scale; SCARED, Screen for Child Anxiety‐Related Emotional Disorders.

The results of partial correlation analysis, which controlled for age, gender, and years of education as covariates, between the clinical symptom‐related scales and the Δβ values of the significantly different channels between the two groups, indicated that the Δβ in the CH4 channel was significantly negatively correlated with total scores of SAD (r = −0.5, *p* = 0.0058), SAD‐social avoidance score (r = −0.5, *p* = 0.0056), and SAD‐social distress score (r = −0.44, *p* = 0.017). Additionally, the Δβ in the CH40 channel was significantly positively correlated with the SCARED‐separation anxiety score (r = 0.41, *p* = 0.032). All data were corrected using FDR correction. See Table 3; Figure 4.

**TABLE 3:** The correlation of clinical symptoms and Δβ in SAD group.

		CH4	CH22	CH34	CH40	CH41
SCARED‐total score	r	−0.227	−0.174	−0.201	0.34	−0.124
	*P*	0.179	0.405	0.335	0.081	0.555
SCARED‐somatization	r	−0.101	−0.083	−0.250	0.314	−0.104
	*P*	0.631	0.695	0.228	0.126	0.622
SCARED‐generalized anxiety	r	−0.265	−0.168	−0.194	0.192	−0.130
	*P*	0.200	0.422	0.352	0.357	0.534
SCARED‐separation anxiety	r	0.050	−0.128	0.090	0.41*	−0.172
	*P*	0.812	0.542	0.670	0.032	0.411
SCARED‐social anxiety	r	−0.290	−0.264	−0.025	0.247	−0.031
	*P*	0.159	0.202	0.904	0.233	0.882
SCARED‐school avoidance	r	−0.113	0.157	−0.258	0.333	−0.107
	*P*	0.590	0.454	0.214	0.103	0.611
SAD‐total score	r	−0.5 **	−0.314	−0.098	0.339	−0.042
	*P*	0.0058	0.126	0.642	0.098	0.844
SAD‐social avoidance	r	−0.5*	−0.382	−0.037	0.37	−0.110
	*P*	0.0056	0.060	0.861	0.054	0.062
SAD‐social distress	r	−0.44*	−0.205	−0.149	0.174	0.035
	*P*	0.017	0.325	0.476	0.404	0.869

Partial correlation coefficients (r) are shown, controlling for age, sex, and education. P‐values are FDR‐corrected for 45 tests (5 channels × 9 clinical measures). *p < 0.05, **p < 0.01 (FDR‐corrected). Abbreviations: SCARED, Screen for Child Anxiety‐Related Emotional Disorders; SAD scale, Social Avoidance and Distress Scale.

## 4 Discussion

To our knowledge, this study is the first application of task‐evoked functional near‐infrared spectroscopy (fNIRS) to compare cortical oxygenated hemoglobin [HbO] dynamics between adolescents diagnosed with SAD and healthy controls (HC), with the aim of preliminarily exploring the potential of neurohemodynamic characteristics for differentiating SAD adolescents. During performance of the verbal fluency task (VFT), individuals with SAD showed significantly greater changes in oxygenated hemoglobin (HbO) concentration within three specific channels relative to healthy controls (HCs). These included channel 22, located in the sensorimotor area (SMA), as well as channel 34 situated in the left ventrolateral prefrontal cortex (vlPFC), and channel 40 in the right vlPFC (Sakakibara et al. 2016; Wang et al. 2022). In contrast, significantly diminished oxygenated hemoglobin (HbO) responses were observed in two channels associated with the dorsolateral prefrontal cortex (dlPFC) and temporal lobe (TL), specifically channels 4 and 41 (Wang et al. 2022). Additionally, the activation in channel 4 was negatively correlated with social avoidance and stress scores, while the activation in channel 40 was positively associated with separation anxiety.

Our research findings reveal that the hemodynamic responses in the vlPFC and SMA of adolescents with SAD are significantly higher than those of healthy individuals. The vlPFC is a region broadly implicated in attention and self‐regulation (Guyer et al. 2012), it plays a role in maintaining anxiety in children (Guyer et al. 2008). Research has indicated that discrepancies in activation between anxious and healthy young adults may be implicated in differing requirements for assessment related to social evaluation. For example, when evaluating social expectations of participants, anxious youth display higher vlPFC activation, which may aid in flexibly selecting appropriate evaluations (Nelson and Guyer 2011). Conversely, when anticipating their own social expectations, vlPFC involvement is lower, potentially reflecting an automatic tendency to predict negative social outcomes (Nelson and Guyer 2011). An fMRI study has indicated that the activation levels of vlPFC in adolescents with SAD are higher than those in healthy adolescents (Guyer et al. 2008). This aligns with our research findings. However, a near‐infrared spectroscopy research study in adult SAD patients indicated lower vlPFC activation than healthy controls (Yokoyama et al. 2015). This difference may be attributed to variations in the age of the study participants, as adults diagnosed with SAD appear to be more susceptible to comorbid depressive disorders, and multiple studies have confirmed that during VFT, patients with depressive disorders show significantly less increase in oxyhemoglobin in the frontotemporal region compared to those with healthy controls (Zhang et al. 2015; Kawano et al. 2016). The increased vlPFC activation observed in our adolescent SAD sample contrasts with some fNIRS findings in adults with SAD reporting decreased vlPFC activation (Yokoyama et al. 2015). This discrepancy suggests a potential developmental stage‐specificity in the neural correlates of SAD. An fMRI study specifically in adolescents with SAD also reported altered vlPFC activity during tasks involving evaluative anticipation (Guyer et al. 2008). Although the task paradigms differ, both findings underscore the significant role of the vlPFC in adolescent SAD. Future studies directly comparing different age cohorts are needed to clarify these developmental neural mechanisms.

The sensorimotor area (SMA) participates in a range of cognitive and motor processes, including motor planning, initiation and inhibition of movements, error monitoring, decision‐making in uncertain conditions, the conscious sensation of movement urge, and the processing of negative feedback (Nachev et al. 2008). Previous research has shown that a larger gray matter volume in the SMA, while enhancing the regulation of motor responses to emotional signals in others, might also render the SMA more susceptible to abnormal modulation by emotional processing regions in response to salient aversive stimuli or high cortical arousal (Voon et al. 2010). The results of a small sample fMRI study have reported increased activation in the SMA during the perception of negative emotional facial expressions such as sadness and fear. These observations suggest a potential involvement of motor‐related cortical and subcortical regions during affective processing, though further research in larger and clinically specific populations is necessary to establish the generalizability of these findings (Aybek et al. 2015). Our study shows increased cortical activation in the SMA, which could be a compensatory phenomenon within the nervous system. Together with the dlPFC, the SMA participates in top‐down control over subcortical motor regions. The heightened activation in the SMA may compensate for impairments in the dlPFC.

We also observed significantly reduced cortical activation in the dlPFC and TL among adolescents with SAD relative to healthy control participants. The dlPFC plays a key role in human cognitive control, particularly in the ability to shift attention (Crone and Steinbeis 2017). However, a significant characteristic of SAD adolescents is an excessive attention to the external environment and inability to focus. Consistent with this, a previous study conducted in a college student population revealed similarly lower cortical activation in the dlPFC of SAD patients compared to HCs (Glassman et al. 2017). Our findings suggest that SAD adolescents may have impaired dlPFC function, which aligns with previous research outcomes (Pantazatos et al. 2014). The dlPFC is central to cognitive control, a function that undergoes significant refinement throughout adolescence (Crone and Steinbeis 2017; Friedman and Robbins 2022). Our finding of reduced dlPFC activation coupled with its negative correlation with social avoidance/distress suggests impaired functional integrity or task‐related recruitment of this region in adolescent SAD. This observation finds a parallel in recent research on brain development in adolescent SAD. A structural covariance study reported age‐dependent alterations in the developmental coordination of cortical‐subcortical networks, implicating prefrontal regulatory circuits (Liu et al. 2024). While the modalities differ, both lines of evidence point towards atypical development within prefrontal regulatory systems in adolescent SAD, which may underlie weakened cognitive control and symptom persistence. The correlation analyses lend further support to these functional interpretations. Specifically, the negative association between dlPFC (CH4) activation and social avoidance/distress symptoms is consistent with its role in cognitive control (Crone and Steinbeis 2017; Friedman and Robbins 2022), suggesting that reduced dlPFC engagement may directly contribute to impaired regulation of social fears. Conversely, the positive link between right vlPFC (CH40) activity and separation anxiety aligns with this region's involvement in emotional reappraisal (Guyer et al. 2012; Goldin et al. 2008; Silvers et al. 2017), possibly reflecting heightened cognitive effort to manage anxiety in separation contexts. These patterns extend prior neuroimaging findings in social anxiety (Glassman et al. 2017; Guyer et al. 2008) by pinpointing their relationship with specific symptom dimensions in adolescents. Neuroimaging studies have indicated that structural and functional alterations in the temporal lobe (TL) may be associated with anxiety‐related phenotypes. For example, one earlier MRI study of a small sample of patients with panic disorder (N = 11) reported reduced TL volume in individuals with high fear sensitivity (Uchida et al. 2003). Our fNIRS findings suggest that there may be potential damage to the TL in adolescent SAD patients. However, further research specifically targeting adolescent SAD populations remains necessary to clarify the neuropathological specificity and developmental trajectory of these alterations.

Result indicate a significant negative correlation between dlPFC activation levels and the severity of both social avoidance and social distress symptoms in adolescents diagnosed with SAD. Avoidance represents a behavioral manifestation, while distress is an emotional response. Our findings suggest that the dlPFC plays a crucial role in both the behavioral and emotional aspects of social interaction in adolescents with SAD, and that social anxiety may be associated with impairments in this region. A recent fMRI study of adolescents with SAD (Hassanvand Amouzadeh 2024) found altered connectivity within the default mode network during self‐evaluation reflection: enhanced excitatory connectivity from the posterior cingulate cortex to the medial prefrontal cortex and increased inhibitory connectivity from the inferior parietal lobule to the medial prefrontal cortex. These changes correspond to the core SAD feature of excessive concern about negative social evaluation. Emerging fMRI‐based neurofeedback provides real‐time brain activity feedback to help regulate emotion‐regulation networks (Lipp and Cohen Kadosh 2020). Adolescents adapting well to this training show increased negative connectivity between the dlPFC and amygdala, enabling better use of cognitive reappraisal, improved mood, and reduced avoidance behaviors (Lisk et al. 2020).

Research conducted with adult populations has demonstrated a correlation between higher levels of social anxiety and reduced activation of the dlPFC in comparison to individuals exhibiting lower levels of social anxiety (Glassman et al. 2017). A slight increase in dlPFC activation has been observed across individuals ranging from low to high anxiety (Glassman et al. 2017). The dlPFC is implicated in cognitive control and the active regulation of emotional states (Friedman and Robbins 2022), and the relationship between dlPFC activation and social anxiety might reflect a shift from effortless thinking to heightened self‐focus in individuals diagnosed with SAD. This suggests that reduced dlPFC activation in individuals with SAD may hinder effective cognitive strategies for regulating emotional responses and behavioral inhibition in social situations, thereby contributing to the persistence of anxiety and avoidance behaviors.

Additionally, our findings show a positive association between vLPFC activity and separation anxiety scores among adolescents diagnosed with SAD. Separation anxiety refers to the anxious emotions experienced when separated from one's closest individuals. Individuals with SAD often experience intensified anxiety due to the unfamiliarity of social environments, feeling tense and fearful when separated from familiar people. A notable characteristic of SAD is over‐reactivity in the prefrontal cortex, particularly on the right side (Engel et al. 2009). When individuals undergo social rejection, the right or bilateral vlPFC is significantly activated (Masten et al. 2009). Our study similarly noted that the higher the activation level in the right vLPFC, the greater the degree of separation anxiety experienced by the individual. Given the role of vlPFC in cognitive reassessment, our research findings may suggest that adolescents with SAD need to make greater efforts to undergo reassessment when separated from familiar individuals. Previous studies also support this view, indicating that anxious youth who lack experience in emotion regulation strategies consume more cognitive resources when attempting effective reassessment (Silvers et al. 2017).

While this study delineates specific cortical activation patterns in adolescent SAD, these functional findings invite consideration of their underlying structural and network bases. The observed hypoactivation of the dlPFC and hyperactivation of the vlPFC/SMA may not only reflect transient task‐related hemodynamics but could be rooted in more stable neurobiological traits. For instance, individual differences in regional gray matter volume within prefrontal‐limbic circuits have been linked to anxiety‐related traits such as intolerance of uncertainty (Suo et al. 2026). Furthermore, the coupling between structural connectivity and spontaneous functional coherence (i.e., structure‐function coupling) is increasingly recognized as a marker of system‐level integrity, with its disruption observed in conditions like depression (Suo et al. 2025). Extending this framework to adolescent SAD, future research could investigate whether the aberrant task‐evoked activation we observed is associated with altered coupling within or between the cognitive control, salience, and default mode networks.

## 5 Limitations

This study has several limitations that should be considered when interpreting the results. First, the relatively small sample size may restrict the statistical power and generalizability of our findings. Although we controlled potential confounds through ANCOVA and FDR correction, the possibility of false‐negative findings cannot be ruled out. Thus, our results are exploratory and require replication in larger samples. Future studies with sufficient power are needed to ascertain whether the identified hemodynamic changes can serve as reliable diagnostic indicators. Second, despite statistically controlling for age, the significant age difference between groups remains an important limitation, as it complicates the separation of disorder‐specific effects from normative developmental changes. In future work, we plan to conduct a follow‐up assessment of the current cohort recruiting age‐matched subgroups. This would allow for a more direct comparison of developmental trajectories and help clarify the specificity of the observed hemodynamic patterns to SAD. Third, the cross‐sectional design, does not allow for inferences regarding neural developmental trajectories or causal relationships between clinical symptoms and neurohemodynamic changes. We intend to conduct longitudinal follow‐up studies to track dynamic functional changes in adolescents with SAD over time, which could help identify potential predictors of illness progression and treatment response. Fourth, symptom severity scales (SCARED, SAD) were administered only to the clinical SAD group. Although healthy controls were screened to exclude any mental disorders, the lack of their quantitative symptom scores limits dimensional analyses across the full sample. Future studies would benefit from collecting such data in control populations for more nuanced comparison. Finally, the current study is limited to cortical hemodynamic measures. Future multimodal imaging work is needed to explore the structural and network‐level correlates of the activation patterns identified here, as discussed above.

## 6 Conclusion

Adolescents with SAD exhibit specific cortical activations, suggesting that hemodynamic changes have the potential to be an auxiliary biomarker for the diagnosis of SAD. Preliminary evidence indicates that certain subregions within the prefrontal cortex (PFC) may be differentially associated with symptom dimensions such as social avoidance and separation anxiety. However, the functional specificity of these regions and their precise contributions to SAD symptomatology require further investigation through experimental designs and larger sample sizes.

## Author Contributions

**Huishan Liu**: writing – original draft, formal analysis, data curation. **Jinru Zhang and Ying Niu**: investigation, data curation. **Weihai Huang and Qiqi Li**: investigation. Zhifen Liu: supervision. Gerard Leavey: writing – review and editing. Gaizhi Li: writing – review and editing, project administration, resources.

## Funding

This study was funded by the National Natural Science Foundation of China Project (82371551, 82001802), Outstanding talents of Shanxi Province (SJYC2024454), Royal Society‐International Exchanges 2024 Cost Share (NSFC)(IES∖R2∖242236), Research Project Supported by Shanxi Scholarship Council of China (2025‐217), Scientific and Technological Innovation Programs of Higher Education Institutions in Shanxi (STIP) (2020L0205), Four “batches” innovation project of invigorating medical through science and technology of Shanxi Province (2023RC010) and the doctoral foundation of Shanxi Medical University (BS201706).

## Ethics Statement

The study was approved by the Medical Ethics Committee of the First Hospital of Shanxi Medical University (Approval No. KYLL‐2023‐276), and written informed consent was obtained from each subject and their parents. All procedures comply with China's Good Clinical Practice (GCP) standards, relevant laws and regulations, as well as the Helsinki Declaration. All participants were native Chinese speakers with a clear understanding, oral ability and can effectively cooperate with the research.

## Data Availability

The datasets used and analyzed during the current study are available from the corresponding author on reasonable request.

## References

[brb371357-bib-0001] Auday, E. S. , and K. E. Pérez‐Edgar . 2019. “Limbic and Prefrontal Neural Volume Modulate Social Anxiety in Children at Temperamental Risk.” Depression and Anxiety 36: 690–700. 10.1002/da.22941. 31373755 PMC6684311

[brb371357-bib-0002] Aybek, S. , T. R. Nicholson , O. O'Daly , F. Zelaya , R. A. Kanaan , and A. S. David . 2015. “Emotion‐Motion Interactions in Conversion Disorder: An FMRI Study.” PLoS ONE 10: e0123273. 10.1371/journal.pone.0123273. 25859660 PMC4393246

[brb371357-bib-0003] Crone, E. A. , and N. Steinbeis . 2017. “Neural Perspectives on Cognitive Control Development During Childhood and Adolescence.” Trends in Cognitive Sciences 21: 205–215. 10.1016/j.tics.2017.01.003. 28159355

[brb371357-bib-0004] Engel, K. , B. Bandelow , O. Gruber , and D. Wedekind . 2009. “Neuroimaging in Anxiety Disorders.” Journal of Neural Transmission 116: 703–716. 10.1007/s00702-008-0077-9. 18568288 PMC2694920

[brb371357-bib-0005] Fehm, L. , A. Pelissolo , T. Furmark , and H. U. Wittchen . 2005. “Size and Burden of Social Phobia in Europe.” European Neuropsychopharmacology 15: 453–462. 10.1016/j.euroneuro.2005.04.002. 15921898

[brb371357-bib-0006] Friedman, N. P. , and T. W. Robbins . 2022. “The Role of Prefrontal Cortex in Cognitive Control and Executive Function.” Neuropsychopharmacology: Official Publication of the American College of Neuropsychopharmacology 47: 72–89. 10.1038/s41386-021-01132-0. 34408280 PMC8617292

[brb371357-bib-0007] Glassman, L. H. , A. T. Kuster , J. A. Shaw , et al. 2017. “The Relationship Between Dorsolateral Prefrontal Activation and Speech Performance‐Based Social Anxiety Using Functional Near Infrared Spectroscopy.” Brain Imaging and Behavior 11: 797–807. 10.1007/s11682-016-9554-1. 27180247

[brb371357-bib-0008] Goldin, P. R. , K. McRae , W. Ramel , and J. J. Gross . 2008. “The Neural Bases of Emotion Regulation: Reappraisal and Suppression of Negative Emotion.” Biological Psychiatry 63: 577–586. 10.1016/j.biopsych.2007.05.031. 17888411 PMC2483789

[brb371357-bib-0009] Guyer, A. E. , V. R. Choate , D. S. Pine , and E. E. Nelson . 2012. “Neural Circuitry Underlying Affective Response to Peer Feedback in Adolescence.” Social Cognitive and Affective Neuroscience 7: 81–92. 10.1093/scan/nsr043. 21828112 PMC3252630

[brb371357-bib-0010] Guyer, A. E. , J. Y. F. Lau , E. B. Mcclure‐Tone , et al. 2008. “Amygdala and Ventrolateral Prefrontal Cortex Function During Anticipated Peer Evaluation in Pediatric Social Anxiety.” Archives of General Psychiatry 65: 1303. 10.1001/archpsyc.65.11.1303. 18981342 PMC2717208

[brb371357-bib-0011] Hassanvand Amouzadeh, M. 2024. “Design and Testing a Model of Some Antecedents of Students Social Anxiety.” Journal of Psychological Studies 20: 119–134.

[brb371357-bib-0012] Henry, J. D. , and J. R. Crawford . 2004. “A Meta‐Analytic Review of Verbal Fluency Performance Following Focal Cortical Lesions.” Neuropsychology 18: 284–295. 10.1037/0894-4105.18.2.284. 15099151

[brb371357-bib-0013] Kawano, M. , T. Kanazawa , H. Kikuyama , A. Tsutsumi , et al. 2016. “Correlation Between Frontal Lobe Oxy‐Hemoglobin and Severity of Depression Assessed Using Near‐Infrared Spectroscopy.” Journal of Affective Disorders 205: 154–158. 10.1016/j.jad.2016.07.013. 27449547

[brb371357-bib-0014] Kessler, R. C. , P. Berglund , O. Demler , R. Jin , K. R. Merikangas , and E. E. Walters . 2005. “Lifetime Prevalence and Age‐of‐Onset Distributions of DSM‐IV Disorders in the National Comorbidity Survey Replication.” Archives of General Psychiatry 62: 593. 10.1001/archpsyc.62.6.593. 15939837

[brb371357-bib-0015] Kir, Y. , D. Sayar‐Akaslan , E. Agtas‐Ertan , et al. 2021. “Cortical Activity During Social Acceptance and Rejection Task in Social Anxiety Disorder: A Controlled Functional Near Infrared Spectroscopy Study.” Progress in Neuro‐Psychopharmacology and Biological Psychiatry 104: 110012. 10.1016/j.pnpbp.2020.110012. 32553940

[brb371357-bib-0016] Lipp, A. , and K. Cohen Kadosh . 2020. “Training the Anxious Brain: Using fMRI‐Based Neurofeedback to Change Brain Activity in Adolescence.” Developmental Medicine & Child Neurology 62: 1239–1244. 10.1111/dmcn.14611. 32638360

[brb371357-bib-0017] Lisk, S. , K. C. Kadosh , C. Zich , S. P. Haller , and J. Y. Lau . 2020. “Training Negative Connectivity Patterns Between the Dorsolateral Prefrontal Cortex and Amygdala Through fMRI‐Based Neurofeedback to Target Adolescent Socially‐Avoidant Behaviour.” Behaviour Research and Therapy 135: 103760. 10.1016/j.brat.2020.103760. 33137695

[brb371357-bib-0018] Liu, J. , S. Xie , Y. Hu , et al. 2024. “Age‐Dependent Alterations in the Coordinated Development of Subcortical Regions in Adolescents With Social Anxiety Disorder.” European Child & Adolescent Psychiatry 33: 51–64. 10.1007/s00787-022-02118-z. 36542201

[brb371357-bib-0019] Ma, Y. , Y. Zou , X. Liu , et al. 2024. “Social Intelligence Mediates the Protective Role of Resting‐State Brain Activity in the Social Cognition Network Against Social Anxiety.” Psychoradiology 4: kkae009. 10.1093/psyrad/kkae009. 38799033 PMC11119848

[brb371357-bib-0020] Masten, C. L. , N. I. Eisenberger , L. A. Borofsky , et al. 2009. “Neural Correlates of Social Exclusion During Adolescence: Understanding the Distress of Peer Rejection.” Social Cognitive and Affective Neuroscience 4: 143–157. 10.1093/scan/nsp007. 19470528 PMC2686232

[brb371357-bib-0021] Mohammadi, M. R. , M. Salehi , A. Khaleghi , et al. 2020. “Social Anxiety Disorder Among Children and Adolescents: a Nationwide Survey of Prevalence, Socio‐Demographic Characteristics, Risk Factors and Co‐Morbidities.” Journal of Affective Disorders 263: 450–457. 10.1016/j.jad.2019.12.015. 31969277

[brb371357-bib-0022] Nachev, P. , C. Kennard , and M. Husain . 2008. “Functional Role of the Supplementary and Pre‐Supplementary Motor Areas.” Nature Reviews Neuroscience 9: 856–869. 10.1038/nrn2478. 18843271

[brb371357-bib-0023] Nelson, E. E. , and A. E. Guyer . 2011. “The Development of the Ventral Prefrontal Cortex and Social Flexibility.” Developmental Cognitive Neuroscience 1: 233–245. 10.1016/j.dcn.2011.01.002. 21804907 PMC3143481

[brb371357-bib-0024] Pantazatos, S. P. , A. Talati , F. R. Schneier , and J. Hirsch . 2014. “Reduced Anterior Temporal and Hippocampal Functional Connectivity During Face Processing Discriminates Individuals With Social Anxiety Disorder From Healthy Controls and Panic Disorder, and Increases Following Treatment.” Neuropsychopharmacology: Official Publication of the American College of Neuropsychopharmacology 39: 425–434. 10.1038/npp.2013.211. 24084831 PMC3870777

[brb371357-bib-0025] Peng, C. , X. Fan , and L. Li . 2003. “The Validity and Reliability of Social Avoidance and Distress Scale in Chinese Students.” Chinese Journal of Clinical Psychology 11: 279–281.

[brb371357-bib-0026] Sakakibara, E. , F. Homae , S. Kawasaki , et al. 2016. “Detection of Resting State Functional Connectivity Using Partial Correlation Analysis: A Study Using Multi‐Distance and Whole‐Head Probe Near‐Infrared Spectroscopy.” Neuroimage 142: 590–601. 10.1016/j.neuroimage.2016.08.011. 27521742

[brb371357-bib-0027] Sareen, J. , B. J. Cox , T. O. Afifi , et al. 2005. “Anxiety Disorders and Risk for Suicidal Ideation and Suicide Attempts: A Population‐Based Longitudinal Study of Adults.” Archives of General Psychiatry 62: 1249. 10.1001/archpsyc.62.11.1249. 16275812

[brb371357-bib-0028] Scaffei, E. , R. Mazziotti , E. Conti , et al. 2023. “A Potential Biomarker of Brain Activity in Autism Spectrum Disorders: A Pilot fNIRS Study in Female Preschoolers.” Brain Sciences 13: 951. 10.3390/brainsci13060951. 37371429 PMC10296408

[brb371357-bib-0029] Scholkmann, F. , S. Kleiser , A. J. Metz , et al. 2014. “A Review on Continuous Wave Functional Near‐Infrared Spectroscopy and Imaging Instrumentation and Methodology.” Neuroimage 85: 6–27. 10.1016/j.neuroimage.2013.05.004. 23684868

[brb371357-bib-0030] Silvers, J. A. , C. Insel , A. Powers , et al. 2017. “vlPFC–vmPFC–Amygdala Interactions Underlie Age‐Related Differences in Cognitive Regulation of Emotion.” Cerebral Cortex 27: 3502–3514. 27341851 10.1093/cercor/bhw073 PMC6059245

[brb371357-bib-0031] Su, L. , K. Wang , F. Fan , Y. Su , and X. Gao . 2008. “Reliability and Validity of the Screen for Child Anxiety Related Emotional Disorders (SCARED) in Chinese Children.” Journal of Anxiety Disorders 22: 612–621. 10.1016/j.janxdis.2007.05.011. 17628391

[brb371357-bib-0032] Suo, X. , L. Chen , G. J. Kemp , and S. Wang . 2026. “Neurostructural Correlates of Intolerance of Uncertainty: Regional and Network‐level Associations With General Psychological Distress.” Neuroimage 325: 121688. 10.1016/j.neuroimage.2026.121688. 41485649

[brb371357-bib-0033] Suo, X. , L. Chen , G. J. Kemp , D. Wu , and S. Wang . 2025. “Aberrant Structural–Functional Coupling of Large‐Scale Brain Networks in Older Women with Subthreshold Depression.” The Journals of Gerontology, Series B: Psychological Sciences and Social Sciences 80: gbaf013. 10.1093/geronb/gbaf013. 39868551

[brb371357-bib-0034] Uchida, H. , and K. Hirao . 2020. “Prefrontal Cortex Hypoactivity Distinguishes Severe From Mild‐to‐Moderate Social Anxiety as Revealed by a Palm‐Sized Near‐Infrared Spectroscopy System.” Journal of Neural Transmission 127: 1305–1313. 10.1007/s00702-020-02228-5. 32638118

[brb371357-bib-0035] Uchida, R. R. , C. M. del‐Ben , A. C. Santos , et al. 2003. “Decreased Left Temporal Lobe Volume of Panic Patients Measured by Magnetic Resonance Imaging.” Brazilian Journal of Medical and Biological Research 36: 925–929. 10.1590/S0100-879X2003000700014. 12845380

[brb371357-bib-0036] Voon, V. , C. Brezing , C. Gallea , et al. 2010. “Emotional Stimuli and Motor Conversion Disorder.” Brain 133: 1526–1536. 10.1093/brain/awq054. 20371508 PMC2859149

[brb371357-bib-0037] Wang, H.‐Y. , X. Hao , R. Lu , et al. 2022. “Effects of Trihexyphenidyl on Prefrontal Executive Function and Spontaneous Neural Activity in Patients With Tremor‐Dominant Parkinson's Disease: An fNIRS Study.” Parkinsonism & Related Disorders 105: 96–102. 10.1016/j.parkreldis.2022.11.012. 36401901

[brb371357-bib-0038] Wang, H.‐Y. , L. Ren , T. Li , et al. 2022. “The Impact of Anxiety on the Cognitive Function of Informal Parkinson's Disease Caregiver: Evidence From Task‐Based and Resting‐State fNIRS.” Frontiers in Psychiatry 13: 960953. 10.3389/fpsyt.2022.960953. 36159948 PMC9492928

[brb371357-bib-0039] Wang, S. , Y. Zhao , X. Wang , et al. 2021. “Emotional Intelligence Mediates the Association Between Middle Temporal Gyrus Gray Matter Volume and Social Anxiety in Late Adolescence.” European Child & Adolescent Psychiatry 30: 1857–1869. 10.1007/s00787-020-01651-z. 33011842

[brb371357-bib-0040] Yokoyama, C. , H. Kaiya , H. Kumano , et al. 2015. “Dysfunction of Ventrolateral Prefrontal Cortex Underlying Social Anxiety Disorder: A Multi‐Channel NIRS Study.” NeuroImage: Clinical 8: 455–461. 10.1016/j.nicl.2015.05.011. 26106570 PMC4474365

[brb371357-bib-0041] Zhang, H. , W. Dong , W. Dang , et al. 2015. “Near‐Infrared Spectroscopy for Examination of Prefrontal Activation During Cognitive Tasks in Patients With Major Depressive Disorder: A Meta‐Analysis of Observational Studies.” Psychiatry and Clinical Neurosciences 69: 22–33. 10.1111/pcn.12209. 24897940

[brb371357-bib-0042] Zhang, X. , X. Suo , X. Yang , et al. 2022. “Structural and Functional Deficits and Couplings in the Cortico‐Striato‐Thalamo‐Cerebellar Circuitry in Social Anxiety Disorder.” Translational Psychiatry 12: 26. 10.1038/s41398-022-01791-7. 35064097 PMC8782859

[brb371357-bib-0043] Zhang, X. , X. Yang , B. Wu , et al. 2023. “Large‐Scale Brain Functional Network Abnormalities in Social Anxiety Disorder.” Psychological Medicine 53: 6194–6204. 10.1017/S0033291722003439. 36330833 PMC10520603

